# Role of Two G-Protein α Subunits in Vegetative Growth, Cell Wall Integrity, and Virulence of the Entomopathogenic Fungus *Metarhizium robertsii*

**DOI:** 10.3390/jof8020132

**Published:** 2022-01-28

**Authors:** Youmin Tong, Hao Wu, Lili He, Jiaojiao Qu, Zhenbang Liu, Yulong Wang, Mingjun Chen, Bo Huang

**Affiliations:** 1 Anhui Provincial Key Laboratory of Microbial Pest Control, Anhui Agricultural University, Hefei 230036, China; tongyoumin@stu.ahau.edu.cn (Y.T.); haowu@stu.ahau.edu.cn (H.W.); 20720507@stu.ahau.edu.cn (L.H.); 5000853@ahau.edu.cn (J.Q.); wyl2019@mtu.edu.cn (Y.W.); 2 School of Life Sciences, University of Science and Technology of China, Hefei 230022, China; lzb1985@ustc.edu.cn

**Keywords:** MrGPA2, MrGPA4, cytoplasm, hyphal growth, pathogenicity

## Abstract

Heterotrimeric G-proteins are crucial for fungal growth and differentiation. The α subunits of heterotrimeric G-proteins play an essential role in controlling signal transduction. However, the function of G-protein α subunits in entomopathogenic fungi remains poorly understood. Two group II Gα subunits (MrGPA2 and MrGPA4) were characterized in the entomopathogenic fungus, *Metarhizium robertsii*. Bioinformatics analysis showed that the relationship between MrGPA2 and MrGPA4 was closer than that of other MrGPAs. Both green fluorescent protein (GFP)-tagged MrGPA2 and MrGPA4 were localized at the cytoplasm. Furthermore, ∆*MrGpa2*∆*MrGpa4* double mutants showed remarkably reduced vegetative growth compared to the wild-type and single-mutant strains, which was accompanied by the downregulation of several growth-related genes, such as *ssk2*, *pbs2*, *stuA*, *hog1*, and *ac*. Only the ∆*MrGpa2*∆*MrGpa4* double mutant was sensitive to Congo red stress. The insect bioassay demonstrated significantly attenuated virulence for the ∆*MrGpa2*∆*MrGpa4* double mutant compared to the wild-type and single-mutant strains. Further analysis indicated that double deletion of *MrGpa2* and *MrGpa4* had no effect on appressorium formation but suppressed the expression levels of several virulence-related genes in the insect hemocoel. These findings demonstrate that *MrGpa2* and *MrGpa4* exhibit functional redundancy and contribute to the vegetative growth, stress tolerance, and pest control potential in *M. robertsii*.

## 1. Introduction

Insect-pathogenic fungi, as a biological control agent that can cause the death of many insects, represent an environmentally friendly alternative to existing insecticides [1]. However, their use has been limited because of their slow killing speed, low sporulation rate, and sensitivity to high temperatures and UV stress. To kill pests, fungal spores first adhere to the pest cuticle. Then, the conidium germinates and penetrates the pest cuticle via mechanical pressure and cuticle-degrading enzymes. At that time, fungal pathogens must dodge acute immune responses to colonize the pest hemocoel. Finally, hyphae multiply within and emerge from the dead pest, and the newly created conidia disperse and infect additional insects if the conditions are favorable [2,3]. An understanding of the molecular mechanisms of its infection, disease development, and stress tolerance is necessary for the commercial application of fungal pathogens.

Heterotrimeric G-proteins, which are critical signaling proteins involved in receptor-mediated signal transduction, are present in all eukaryotic cells [4]. The G-proteins consisting of α, β, and γ subunits are coupled to the G-protein-coupled receptor (GPCR) for transferring environmental signals [5]. G-proteins are crucial for the regulation of cell function, division, and differentiation. In filamentous fungi, they contribute to hyphal growth, conidiation, conidial germination, stress resistance, and virulence [6,7,8,9].

G-protein α subunits can be classified into four groups (Gα_i_, Gα_s_, Gα_q_, and Gα_12_) in mammalian species, and only two kinds of Gα subunits (canonical Gα and Extra-large Gα_s_) exist in plants [4]. In yeast, two Gα subunits (GPA1 and GPA2) have been identified [10]. In particular, Gα subunits of the filamentous fungi are classified into three major groups: group I, group II, and group III [11]. The groups I and III Gα proteins contain a consensus myristylation site (MGXXXS) in the N terminus and a pertussis toxin-labeling site (CXXX) at the C termini of group I Gα proteins, but group II Gα proteins do not contain either site [11]. Based on their different structures, the functions of group II Gα proteins are diverse. The role of group II Gα proteins has been characterized in some fungi. For instance, the deletion of GNA-2 in *Neurospora crassa* intensified a reduction in mass accumulation on poor carbon sources in ∆*gna-1* or ∆*gna-3* strains [12]. In *Magnaporthe grisea*, disruption of *magC* resulted in a reduction in conidia [8]. ∆*bcg-2* of *Botrytis cinerea* was only slightly reduced in pathogenicity [13]. Disruption of *fga*3 in *Fusarium oxysporum* f. sp. *cucumerinum* led to higher thermal resistance, possibly through the cAMP-dependent protein kinase A pathway [14]. However, the functions of the group II Gα proteins in entomopathogenic fungi are still unknown.

An entomopathogenic model fungus, *Metarhizium robertsii*, has been well developed as one of the most environmentally friendly insecticides [2]. Genes encoding four Gα subunits, *MrGpa1* (EFZ00892.1), *MrGpa2* (EFY98464.1), *MrGpa3* (EFY99066.2), and *MrGpa4* (EFZ00060.2), have been identified in *M. robertsii* [9]. We have previously shown that MrGPA1, belonging to group I, contributes to conidiation, stress resistance, virulence, and intracellular cAMP levels, while *MrGpa3*, which was identified as a group III Gα subunit, plays similar biological roles in the fungus [9]. MrGPA2 and MrGPA4 of *M. robertsii* are orthologs of *N. crassa* GNA-2; However, there is no report on the function of MrGPA2 and MrGPA4 in entomopathogenic fungi.

## 2. Materials and Methods

### 2.1. Fungal Strains and Culture

The wild-type (WT) strain *M. robertsii* ARSEF 23 (ATCC No.MYA-3075) was isolated from Conoderus sp. in North Carolina, USA, in 1961. The fungus and all of the mutant strains were maintained on potato dextrose agar (PDA, 20% potato, 2% glucose, and 2% agar, *w*/*v*) at 25 °C under dark conditions for 10 days [15]. After 10 days of incubation, conidia were harvested by vortex-mixing in 0.05% (*v*/*v*) Tween-80, filtrated through sterile non-woven fabric, and washed with sterile water.

### 2.2. Sequence Analysis

To determine the phylogenetic relationship among MrGPA2, MrGPA4, and their orthologues, the amino acid sequences of those functionally characterized GPA subunits of different fungi were downloaded from the National Center for Biotechnology Information (https://www.ncbi.nlm.nih.gov/, accessed on 20 September 2021). A neighbor-joining tree was constructed using the program MEGAX (https://www.megasoftware.net/, accessed on 20 September 2021).

### 2.3. Subcellular Localization of MrGPA2 and MrGPA4

To investigate the subcellular localization of MrGPA2 and MrGPA4, two genes were fused with a GFP tag and inserted into the plasmid pDHt-SK-*bar* (kindly provided by Dr. Chengshu Wang; the vector conferred resistance against glufosinateammonium) containing a strong promoter and a terminator, which were transformed into *M. robertsii* and screened as previously described [16]. The subcellular localization of MrGPA2-GFP and MrGPA4-GFP fusion proteins in conidia and mycelia were visualized using confocal microscopy (LSCM, Zeiss LSM980, Zeiss Gruppe, Oberkochen, Baden-Wurttemberg, German).

### 2.4. Gene Deletion and Complementation

Targeted gene disruption of *MrGpa2* and *MrGpa4* was performed by homologous recombination via *Agrobacterium tumefaciens* transformation as previously described [9]. In brief, the 5′ and 3′ flanking regions of *MrGpa2* and *MrGpa4* were inserted into the pDHt-SK-*bar*, and then the vectors pDHt-*MrGpa2*-*bar* and pDHt-*MrGpa4*-*bar* were obtained for fungal transformation. For double-gene deletion, the 5′ and 3′ flanking regions of *MrGpa4* were inserted into the pDHt-SK-*ben* vector (containing benomyl resistance gene), and the product was transformed into Δ*MrGpa2* strain and screened as previously mentioned.

For mutant complementation, the entire *MrGpa2* and *MrGpa4* genes were amplified together with their upstream and downstream regions and then inserted into the vector pDHt-SK-*ben* to produce the vectors pDHt-cp*MrGpa2*-*ben* and pDHt-cp*MrGpa4*-*ben* for fungal transformation. Next, all of transformants were verified by DNA sequencing. All the primers used in this study are listed in Supplementary Materials, Table S1.

### 2.5. Phenotype Assays

To assess conidiation capacity, 30 µL of conidial suspension (1 × 10^6^ conidia/mL) of WT, ∆*MrGpa2*, ∆*MrGpa4*, ∆*MrGpa2*∆*MrGpa4*, cp∆*MrGpa2*, and cp∆*MrGpa4* strains was evenly spread on PDA medium and incubated for 14 days at 25 °C. These samples were then placed in 30 mL of 0.05% Tween 80 to release conidia by vortex-mixing, and these conidial suspensions were used for assessment of conidial concentration with a hemocytometer, followed by conversion of the number of conidia per square centimeter of the colony.

The vegetative growth assays of the WT, ∆*MrGpa2*, ∆*MrGpa4*, ∆*MrGpa2*∆*MrGpa4*, cp∆*MrGpa2*, and cp∆*MrGpa4* strains were carried out on PDA, SDAY, and 1/4 SDAY (1/4 dilution of SDAY) media. For this, samples of conidial suspensions (1 µL; 1 × 10^7^ conidia/mL) were point-inoculated on each plate and incubated at 25 °C in the dark for 10 days. The colony diameters were then measured.

To determine the growth rates on Czapek agar (CZA, 3% sucrose, 0.3% NaNO_3_, 0.1% K_2_HPO_4_, 0.05% KCl, 0.05% MgSO_4_, 0.001% FeSO_4_,and 2% agar), amended CZAs containing different carbon or nitrogen sources were prepared by deleting 3% sucrose or 0.3% NaNO_3_; replacing the sole carbon source (3% sucrose) with 3% glucose, trehalose, lactose, fructose, maltose, mannitol, sorbitol, glycerol, ethanol, sodium acetate (NaAc), or oleic acid; and replacing the sole nitrogen source (0.3% NaNO_3)_ with 0.3% NH_4_Cl or one of 20 amino acids, followed by incubation at 25 °C for 10 days. The colony diameters were then measured.

To analyze the chemical tolerance, conidial suspensions (1 μL; 1 × 10^7^ conidia/mL) of strains were cultured on PDA plates supplemented with NaCl (0.5 M), H_2_O_2_ (2 mM), or Congo red (2 mg/mL) in the dark at 25 °C for 10 days. The colony diameter was measured, and the relative inhibition rate was calculated.

To assay the heat tolerance, experiments were conducted on PDA plates. Conidial suspensions (5 × 10^6^ conidia/mL) of strains were incubated in a water bath at 42 °C or 28 °C (as control) for 1 h, and then 10 μL of the suspension was incubated at 25 °C in the presence of the medium. Germination was observed with Olympus BX 51(Tokyo, Japan)) at 12, 16, 20, and 24 h. Conidia were considered germinated when the length of the germ tube projecting from it reached the half length of the conidia. A total of 300 hundred conidia were counted per plate, and the relative germination rates were calculated to allow comparisons of the number of germinated conidia with and without heat stress.

To evaluate fungal tolerance to ultraviolet B (UV-B) light, heat tolerance assays were conducted as previously mentioned. These plates with conidia were exposed to an irradiance of 312 nm wavelength at 100 μJ cm^−2^ using HL-2000 Hybrilinker (UVP, Upland, CA, USA). Relative percent germination was assessed using the aforementioned methods.

For virulence assays, the tests were performed using *Galleria mellonella* larvae as previously described [9]. Briefly, conidial suspensions (1 × 10^7^ conidia/mL) harvested from a 10-day-old PDA culture were collected, and the larvae were separately immersed in each conidial suspension for 90 s, followed by incubation at 25 °C. All treated cohorts were performed in triplicate, with 18 larvae in each group, and monitored every 12 h to record survival, using the SPSS software to calculate the median lethal time (LT_50_) by probit analysis.

For appressorium formation assay, 1 mL of conidial suspension (1 × 10^6^ conidia/mL) in MMGly (minimal medium amended with 1% glycerol) was spread on a sterile plastic Petri dish (3.5 cm diameter), followed by 24 h incubation at 25 °C for 24 h. The appressorium formation rates were assessed as described previously [17].

### 2.6. Quantitative RT-PCR (RT-qPCR)

To analyze the transcriptional profiling of phenotype-related genes, WT, ∆*MrGpa2*∆*MrGpa4*, controls, and two single-mutant strains were cultured on PDA plates for 10 days, or *G. mellonella* larvae were infected by six strains for 48 h. Then, the mycelia or larval hemocoel were harvested and milled in liquid nitrogen for RNA extraction. Total RNA was isolated using the TRIzol reagent (Invitrogen, Foster City, CA, USA). cDNA was used as a template for RT-qPCR using a HiScript III 1st strand cDNA synthesis kit (Vazyme, Nanjing, China). Three biological replicates were analyzed for each treatment. qRT-PCR analysis was performed using the CFBR96TM Real-Time PCR System (Bio-Rad, Hercules, CA, USA) and AceQ qPCR SYBR Green Master Mix (Vazyme, Nanjing, China). The primers used are listed in Supplementary Material, Table S2. The *gpd* gene (MAA_07675, encoding glyceraldehyde 3-phosphate dehydrogenase) was used as an internal control, and relative gene expression was determined by normalizing the expression of each gene to GAPDH. The data were analyzed using the 2^−ΔΔCt^ method [18].

### 2.7. Statistical Analysis

Each experiment was performed in triplicate. All data were analyzed using GraphPad Prism version 7.0 and SPSS v23.0 software (SPSS Inc., Chicago, IL, USA). Kolmogorov—Smirnov test and Levene’s test were used to confirm that the data followed normality and homoscedasticity, respectively. Then, data (mean  ±  SE) from different experimental groups were analyzed using Student’s *t*-test, one- or two-way analysis of variance (ANOVA) followed by a least significant difference (LSD) test. *p* < 0.05 and *p* < 0.01 were considered significant and extremely significant, respectively. * *p* < 0.05, ** *p* < 0.01.

## 3. Results

### 3.1. Characteristics of MrGPA2 and MrGPA4 from M. robertsii

**Figure 1:**
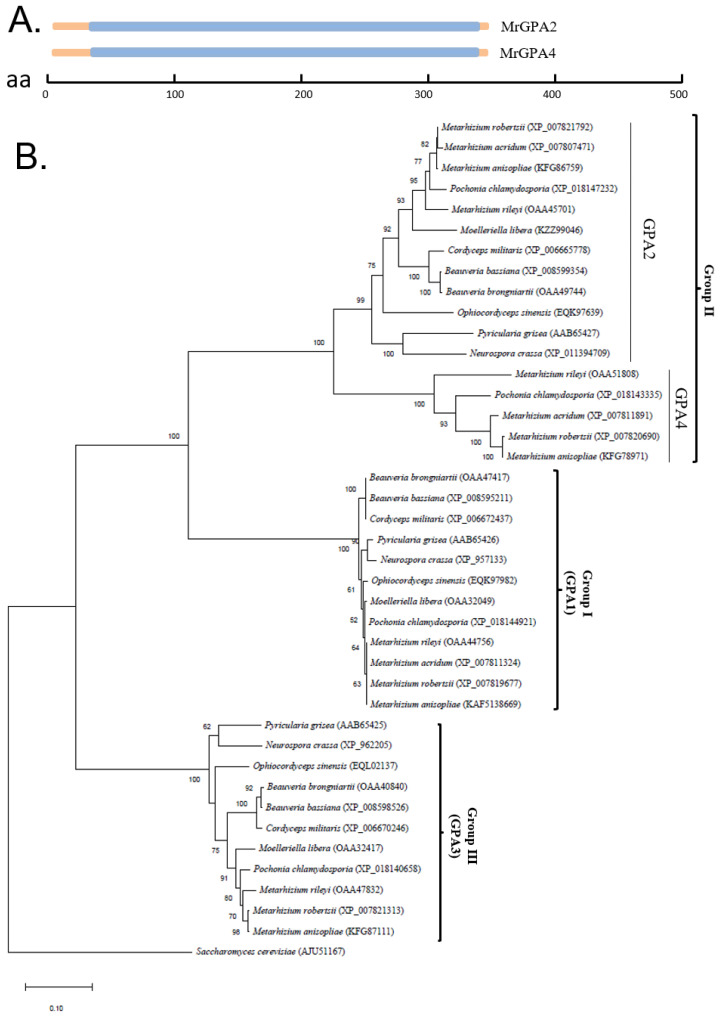
Phylogenetic analysis of four G-proteins in *Metarhizium robertsii*. (**A**) Conserved domain analysis of MrGPA2 and MrGPA4. The guanine nucleotide-binding domains of the GPAs are shown in blue. aa, amino acid. (**B**) Phylogenetic relationship analysis of four G-proteins in *M. robertsii* and their orthologs in different fungi by the neighbor-joining method. Four G-proteins of *M. robertsii* and their related proteins are given in brackets following each fungal name.

We identified two *N. crassa* GNA-2 homologs, EFY98464.1 and EFZ00060.2, in *M. robertsii*, and named them MrGPA2 and MrGPA4, respectively. Bioinformatics analysis showed that MrGPA2- and MrGPA4-encoded proteins had 354 amino acids and 353 amino acids, respectively. Conserved domain analysis revealed that *MrGpa2* and *MrGpa4* contained the guanine nucleotide-binding site of heterotrimeric G-protein (34-347-aa protein and 34-348-aa protein) (Figure 1A).

The Gα sequences from the filamentous fungi were selected for phylogenetic analysis using *Saccharomyces cerevisiae* as an outgroup and were divided into three main clades including the group I (MrGPA1 *M. robertsii*), group II (MrGPA2 and MrGPA4 *M. robertsii*), and group III (MrGPA3 *M. robertsii*) clans (Figure 1B). Moreover, these MrGPA2 and MrGPA4 homologs were grouped together, and the relationship between MrGPA2 and MrGPA4 was closer than that of other MrGPAs. Intriguingly, phylogenetic analysis indicated that *M. robertsii* MrGPA4 homologs were only found in members of the genus *Metarhizium* and its sister genus *Pochonia*, whereas *M. robertsii* MrGPA2 homologs existed in filamentous fungi. Based on the relationship between the two GPAs and their distribution in filamentous fungi, MrGPA4 is likely an additional copy of MrGPA2.

### 3.2. MrGPA2 and MrGPA4 Are Located in Cytoplasm

**Figure 2:**
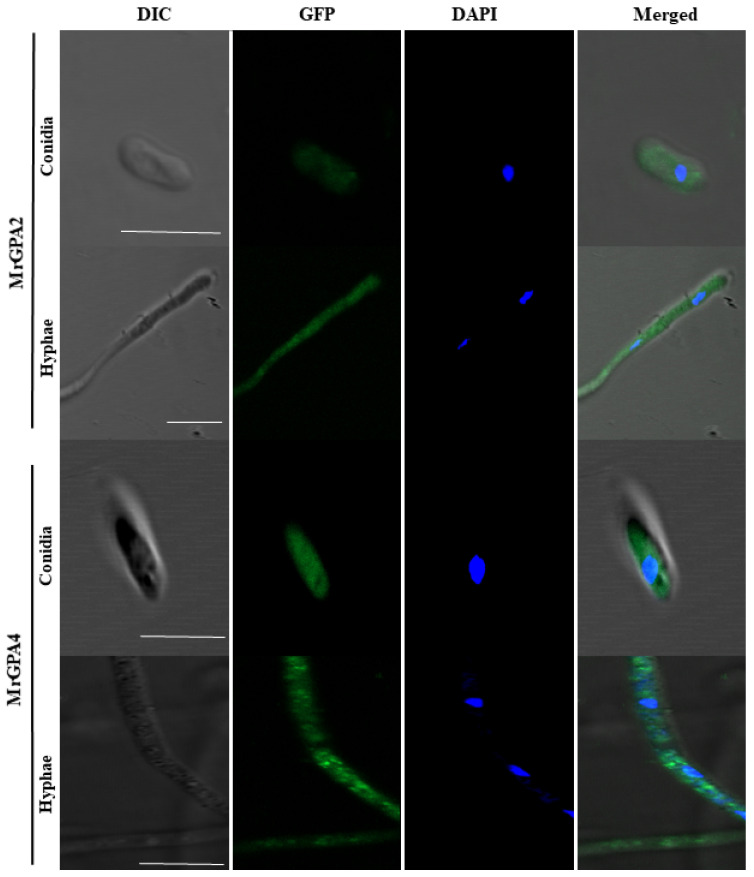
Localization of MrGPA2 and MrGPA4. The localization of MrGPA2 and MrGPA4 in hyphae and conidia, showing that MrGPA2 and MrGPA4 are located in the cytoplasm with the GFP fluorescence signal, scale bars: 10 μm.

To assess the subcellular localization of MrGPA2 and MrGPA4, we generated *MrGpa2*-*gfp* and *MrGpa4*-*gfp* strains for fluorescence observation (Supplementary Figure S1A–C). As shown in the LSCM images in Figure 2, the green fluorescence signals of MrGPA2 and MrGPA4 or 4,6-diamidino-2-phenylindole (DAPI)-stained nuclei were detected, but they were only observed in the cytoplasm, either in hyphae or in conidia, which both exhibited the cytoplasmic localization of MrGPA2 and MrGPA4.

### 3.3. Gene Knockout and Complementation

To further investigate the function of *MrGpa2* and *MrGpa4* in *M. robertsii*, ∆*MrGpa4*, ∆*MrGpa4*, double-knockout mutants ∆*MrGpa2*∆*MrGpa4* were generated and detected by PCR amplification. (Supplementary Figure S1A,B). PCR analysis revealed that the one 286-bp fragment corresponding to the partial *MrGpa2* gene sequence was not detected in the ∆*MrGpa2* and ∆*MrGpa2*∆*MrGpa4* strains, and the one 212-bp fragment corresponding to the partial *MrGpa4* gene sequence was not found in the ∆*MrGpa4* or ∆*MrGpa2*∆*MrGpa4* strains. In addition, a partial 434-bp *bar* gene fragment was present in all transformants, and a partial 1993-bp *ben* gene fragment was detected in ∆*MrGpa2*∆*MrGpa4*, cp∆*MrGpa2,* and cp∆*MrGpa4*. These observations indicated the presence of deletion mutants (∆*MrGpa4*, ∆*MrGpa2*, and ∆*MrGpa2*∆*MrGpa4*) and complementation strains (cp∆*MrGpa2* and cp∆*MrGpa4*).

### 3.4. ∆MrGpa2∆MrGpa4 Strain Leads to a Decrease in Vegetative Growth but Had No Effect on Conidiation

**Figure 3:**
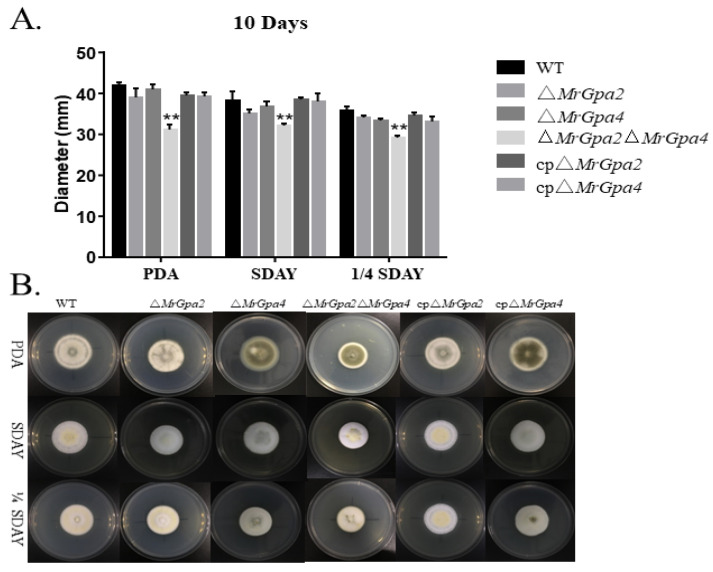
*MrGpa2* and *MrGpa4* contribute to vegetative growth but are not involved in conidiation. (**A**) Colony diameters of six fungal strains on PDA, SDAY, and 1/4SDAY media. (**B**) Colony phenotype of six fungal strains. ** *p* < 0.01.

We assessed the effect of MrGPAs’ disruption on vegetative growth. The single mutants of *MrGpa2* or *MrGpa4* had no influence in vegetative growth, but the double mutants affected vegetative growth. The growth rate of the ∆*MrGpa2*∆*MrGpa4* mutant was significantly reduced by 26% (*F*_5, 23_ = 41.2, *p* < 0.01), 16% (*F*_5, 23_ = 15.5, *p* < 0.01), and 18% (*F*_5, 23_ = 36.9, *p* < 0.01) in PDA, SDAY, and 1/4 SDAY plates, respectively, compared to the WT strain (Figure 3A,B).

**Figure 4:**
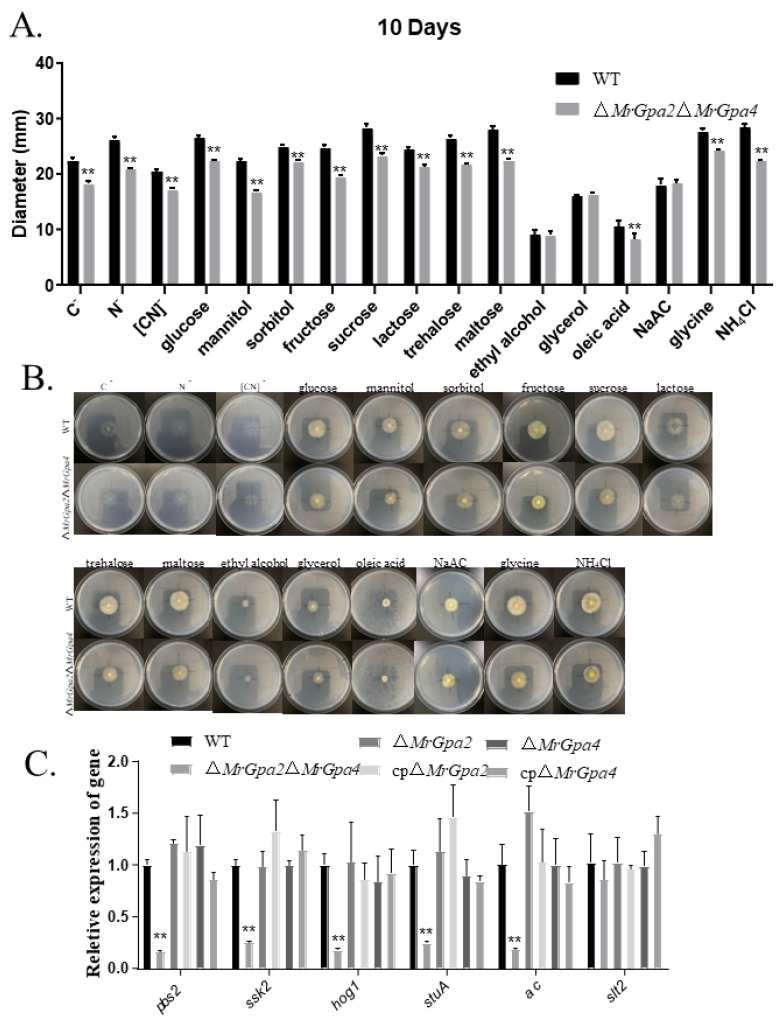
*MrGpa2* and *MrGpa4* affect hyphal growth in CZAs plates. (**A**) Colony diameters of WT and ∆*MrGpa2*∆*MrGpa4* strains were estimated on the plates of CZA and modified CZAs and in the absence of carbon (C–), nitrogen (N–), or both ([CN]–). (**B**) Colony phenotype of WT and ∆*MrGpa2*∆*MrGpa4* strains. (**C**) The expression of six growth-associated genes in WT and ∆*MrGpa2*∆*MrGpa4* strains. ** *p* < 0.01.

To further verify the effect of nutrient utilization on mutant growth in mixed-media CZAs amended with different carbon (sugars/non-sugars) or nitrogen (inorganic/organic) sources, colony diameters were assessed in the ∆*MrGpa2*∆*MrGpa4* and WT strains. The ∆*MrGpa2*∆*MrGpa4* mutant grew significantly slower than the WT strains on CZA media amended with sugar carbon sources such as glucose (*t* = 24.8, *p* < 0.01), trehalose (*t* = 23.5, *p* < 0.01), lactose (*t* = 13, *p* < 0.01), maltose (*t* = 19.9, *p* < 0.01), mannitol (*t* = 26.2, *p* < 0.01), or sorbitol (*t* = 12.9, *p* < 0.01), and CZAs media amended with the nitrogen sources with NH_4_Cl (*t* = 25.8, *p* < 0.01), or glycine (*t* = 15.2, *p* < 0.01) (Figure 4A,B). In contrast, colony size was not significantly different between ∆*MrGpa2*∆*MrGpa4* and the control strain grown on CZA media supplemented with a non-sugar carbon source such as ethyl alcohol (*t* = 0.3, *p* = 0.75), glycerol (*t* = 0.9, *p* = 0.31), and NaAC (*t* = 0.7, *p* = 0.53). Moreover, the expression of six growth-related genes in *M. robertsii* was examined using RT-qPCR to explore the roles of the two genes in fungal vegetative growth. The ∆*MrGpa2*∆*MrGpa4* strain demonstrated markedly reduced expression of *ssk2* (MAA-04290, *F*_5, 12_ = 18.9, *p* < 0.01), *pbs2* (MAA-00856, *F*_5, 12_ = 14.2, *p* < 0.01), *stuA* (MAA-02988, *F*_5, 12_ = 6.2, *p* < 0.01), *hog1* (MAA-05126, *F*_5, 12_ = 12.0, *p* < 0.01), and *ac* (MAA-07239, *F*_5, 12_ = 11.7, *p* < 0.01) (Figure 4C). Collectively, these data indicate that *MrGpa2* and *MrGpa4* are involved in carbon/nitrogen metabolism and affect fungal vegetative growth.

**Figure 5:**
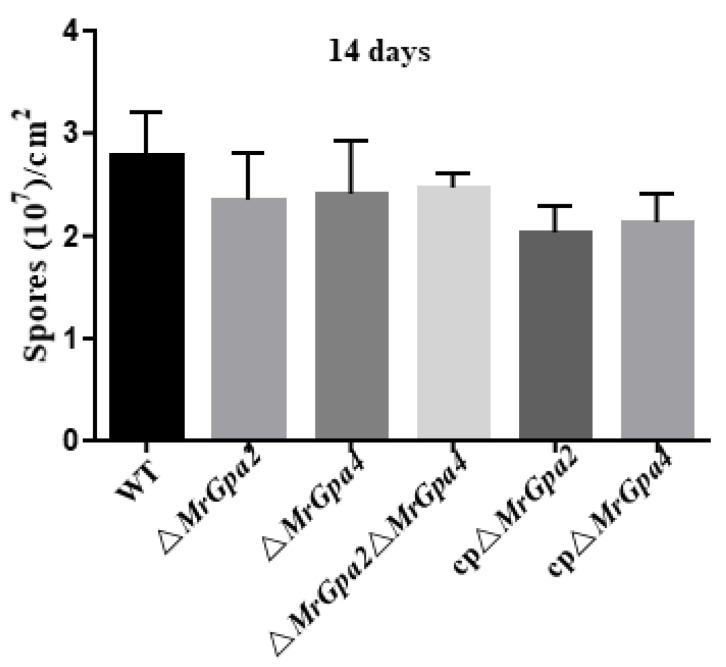
The conidial yield of six strains on PDA plates at 25 °C for 14 days.

In addition, the conidial yield was also assessed for the three mutants, which confirmed that the ∆*MrGpa2*, ∆*MrGpa4*, and ∆*MrGpa2*∆*MrGpa4* strains were not required for conidial yield (Figure 5).

### 3.5. Deletion of Both MrGpa2 and MrGpa4 Enhanced Cell Wall Integrity

**Figure 6:**
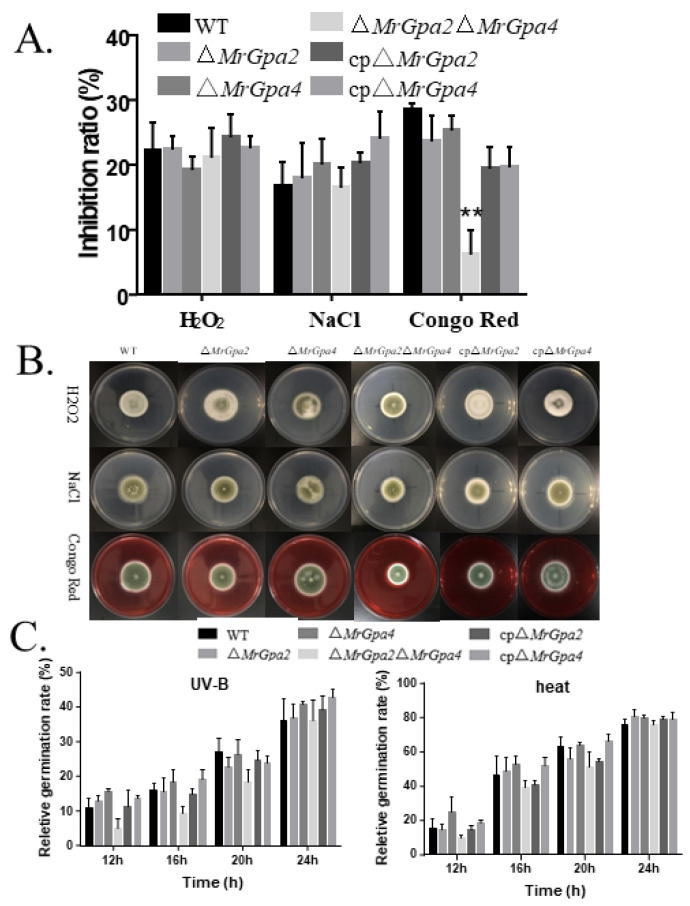
*MrGpa2* and *MrGpa4* affect cell wall integrity. (**A**) Inhibition ratio of six fungal strains on PDA medium containing NaCl (0.5 M), H2O2 (10 mM), or Congo red (2 mg/mL). (**B**) Colony phenotype of WT and ∆*MrGpa2*∆*MrGpa4* strains. (**C**) Conidial relative germination of six strains on PDA medium 12, 16, 20, and 24 h after exposure to UV-B irradiation stress or heat stress at 42 °C. ** *p* < 0.01.

To examine whether the ∆*MrGpa2*, ∆*MrGpa4*, and ∆*MrGpa2*∆*MrGpa4* strains showed an altered response to chemical stress, mycelial growth was observed on a PDA medium containing NaCl, H_2_O_2_, or Congo red (Figure 6A,B). On PDA plates containing Congo red, the ∆*MrGpa2*∆*MrGpa4* strain showed altered mycelial diameter and a 78.3% reduction in the inhibition ratio compared to that of the WT strain (*F*_5, 21_ = 33.9, *p* < 0.01), which indicates the enhanced cell wall integrity of the double mutant. However, no effects on osmotic or antioxidant stress were observed in the ∆*MrGpa2*, ∆*MrGpa4*, or ∆*MrGpa2*∆*MrGpa4* strains.

Furthermore, to confirm the role of *MrGpa2* and *MrGpa4* in heat and UV-B tolerance, conidial germination rates were calculated at 12, 16, 20, and 24 h after exposure to heat or UV-B irradiation stress (Figure 6C). Compared with the WT and CP strains, the conidial germination rates in the three mutants were not significantly different.

### 3.6. ∆MrGpa2∆MrGpa4 Strain Displayed an Increased Survival Rate of the Larvae in Bioassays

**Figure 7:**
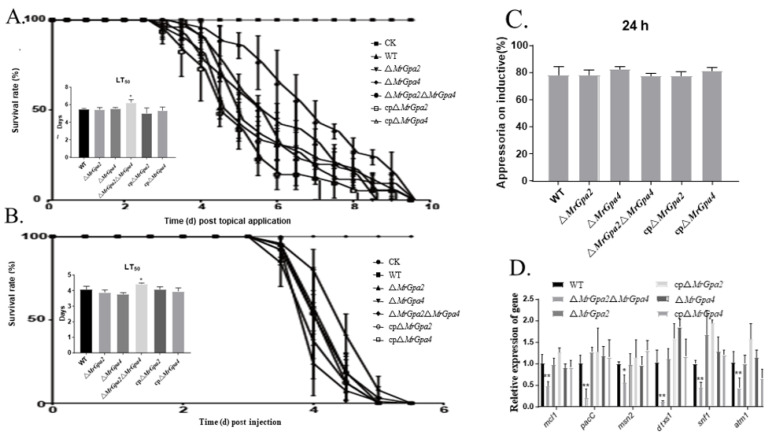
*MrGpa2 and MrGpa4* impact fungal virulence. (**A**) Survival rate and LT_50_ (days) of *G.mellonella* after topical application of conidial suspensions in six fungal strains. The control insects were treated with sterile water. (**B**) Survival rate and LT_50_ (days) of *G.mellonella* after injection with conidial suspensions in six fungal strains. The control insects were treated with sterile water. (**C**) The appressorium formation rates of six fungal strains. (**D**) Expression levels of virulence-associated genes. * *p* < 0.05, ** *p* < 0.01.

To investigate the role of *MrGpa2* or *MrGpa4* in virulence, the mortality rate of *G. mellonella* larvae caused by each mutant was assessed using bioassays (topical and injection). After topical application, the differences were significant between the WT (LT_50_ = 5.52 ± 0.06 days) and ∆*MrGpa2*∆*MrGpa4* strains (LT_50_ = 6.24 ± 0.20 days) (*F*_5, 21_ = *p* < 0.05) (Figure 7A). However, no significant difference was observed for ∆*MrGpa2* (LT_50_ = 5.45 ± 0.18 days) or ∆*MrGpa4* (LT_50_ = 5.59 ± 0.06 days) compared with WT. Similarly, after the intrahemocoel injection, the insect groups infected with the ∆*MrGpa2*∆*MrGpa4* strain exhibited an LT_50_ of 4.41 ± 0.05 days, with a significant attenuation of virulence, while the WT, control, and two single-mutant strains displayed LT_50_ values of 4.08 ± 0.10, 4.09 ± 0.06, 3.96 ± 0.11, 3.88 ± 0.07, and 3.76 ± 0.04 days, respectively (*F*_5, 21_ = 6.9, *p* < 0.05) (Figure 7B). The ∆*MrGpa2*∆*MrGpa4* strain displayed an increase in LT_50_ and thus a significant difference in virulence, but the two single mutants demonstrated virulence comparable to the WT strain after both topical infection and injection of fungal spores into *G. mellonella*.

Furthermore, the appressorium formation rates were not remarkably different among the WT, ∆*MrGpa2*∆*MrGpa4*, control, or the two single-mutant strains, suggesting that *MrGpa2* and *MrGpa4* have no effect on fungal appressorium formation, which is required for cuticle penetration (Figure 7C).

Moreover, we investigated the transcriptional level of virulence-related genes in the insect hemocoel using RT-qPCR. Indeed, the transcriptional levels of *mcl1* (a collagen-like protein, *F*_5, 12_ = 9.5, *p* < 0.01), *pacC* (a pH regulatory protein, *F*_5, 12_ = 5.2, *p* < 0.01), *msn2* (a transcription factor, *F*_5, 12_ = 3.2, *p* < 0.05), *dtxS1* (a nonribosomal peptide synthetase, *F*_5, 12_ = 14.2, *p* < 0.01), *snf1* (encoding a sucrose-nonfermenting protein kinase, *F*_5, 12_ = 10.5, *p* < 0.01), and *atm1* (acid trehalose, *F*_5, 12_ = 7.6, *p* < 0.01) were markedly downregulated in the ∆*MrGpa2*∆*MrGpa4* strain, compared with the WT, control, and two single-mutant strains (Figure 7D) [19,20,21,22,23,24]. Therefore, *MrGpa2* and *MrGpa4* contribute to fungal virulence by affecting colonization and fungal cell growth in the insect hemocoel.

## 4. Discussion

As previously reported, G-proteins regulate a variety of fungal physiological processes, including conidiation, vegetative growth, stress tolerance, appressoria formation, and virulence [5,9,25]. In this study, we identified two group II Gα proteins, MrGPA2 and MrGPA4, in *M. robertsii*, both of which are involved in the vegetative growth, cell wall integrity, and virulence of *M. robertsii*, although the two single mutants did not display any defect in phenotypic characteristics.

Fungal Gα-proteins are classified into three major groups: group I, group II, and group III. In contrast to yeast, which contains two Gα proteins, most characterized filamentous fungi possess three Gα proteins that are members of distinct groups, including *Magnaporthe grisea*, *Neurospora crassa*, and *Fusarium oxysporum* f. sp. *cucumerinum* [4,7,8,12,14]. However, four Gα proteins have been identified in the genus *Metarhizium* and its sister genus *Pochonia*. Further analysis showed that group II consisted of MrGPA4 and MrGPA2. In this study, we found that MrGPA2 and MrGPA4 were functionally redundant in *M. robertsii*.

To transmit extracellular signals to intracellular effectors, G-proteins are localized to the plasma membrane [26]. However, in recent years, G-proteins have been identified in many other intracellular organelles, such as the mitochondria, Golgi apparatus, endoplasmic reticulum, and nucleus [27]. In our previous *Metarhizium* GPA study, MrGPA1 and MrGPA3 were found to be localized in the mitochondria and vacuoles, respectively [9]. However, in this study, both MrGPA2 and MrGPA4 were found to be localized in the cytoplasm, unlike the *S. cerevisiae* GPA2 embedded in the plasma membrane [10]. We speculate that the function of MrGPA2 and MrGPA4 differs from that of other fungal GPAs.

In this study, the ∆*MrGpa2*∆*MrGpa4* double mutant showed inhibited growth in PDA, SDAY, 1/4 SDAY, and CZAs plates. Further growth analyses indicated that the double-deletion mutant was involved in carbon and nitrogen metabolism. In other filamentous fungi, the role of the group II Gα subunits is not related to growth, such as *N. crassa*, *M. grisea*, *B. cinerea*, and *F. oxysporum* f. sp. *cucumerinum* [11,13,14]. Thus, the relative importance of group II Gα subunits for colony growth may vary among fungal groups.

The LT_50_ values of the ∆*MrGpa2*∆*MrGpa4* strain after topical application and injection were higher than those of the WT strain. However, appressorium formation in the ∆*MrGpa2*∆*MrGpa4* double mutant was unaffected. In addition, the double-deletion mutant was found to be involved in carbon and nitrogen metabolism. Thus, the double deletion of *MrGpa2* and *MrGpa4* led to a statistically significant increase in the survival rate of *G. mellonella* larvae, which was probably due to the slower growth of the ∆*MrGpa2*∆*MrGpa4* strain in *G. mellonella* hemocoel. This hypothesis was subsequently confirmed by the decrease in the expression of *atm1* in the ∆*MrGpa2*∆*MrGpa4* strain. In addition, the decrease in virulence of the ∆*MrGpa2*∆*MrGpa4* strain might be partly due to defects in colonization, owing to the decrease in the expression of *mcl1* (evading insect immune responses) [20,24]. Similarly, the group II Gα subunit of ∆*bcg-2* in *B. cinerea* is slightly reduced in pathogenicity, and BCG2 is also necessary for a normal colonization rate [13].

Gα-proteins are extremely important in heterotrimeric G-protein signaling and regulate the growth and differentiation of pathogenic fungi. In *Magnaporthe grisea*, Gα subunits MAGA and MAGB are directly modulated by RGS1, which physically interacts with GPCR PTH11 in vivo to activate MAPK cascades in appressorium formation [28]. The loss of G-protein-coupled receptor K (GPRK) causes defects in radial growth in *M. robertsii* [29]. In *M. robertsii*, the deletion of GPCR *MrGpr8* significantly downregulates two Gα subunit genes during appressorium formation [30]. In this study, we found that the deletion of two Gα subunits (*MrGpa2* and *MrGpa4*) resulted in the defect of fungal growth rather than appressorium formation. We also found that relative to the WT, the MAPK components *ssk2*, *pbs2*, and *hog1* were significantly downregulated in ∆*MrGpa2*∆*MrGpa4*. Thus, Gα subunits act as downstream GPCRs and upstream components of MAPK during appressorium differentiation and fungal growth.

Overall, this study revealed that both MrGPA2 and MrGPA4, belonging to group II Gα, are functionally redundant and required for vegetative growth, cell wall integrity, and virulence.

These findings broaden our understanding of the functions of G-proteins in entomopathogenic fungi and provide new insights into the genetic improvement of *M. robertsii* for pest control.

## Data Availability

Not applicable.

## Supplementary Materials

The following are available online at https://www.mdpi.com/article/10.3390/jof8020132/s1. Figure S1: Gene deletion, complementation, and construction of GFP fusion vector of *MrGpa2* and *MrGpa4* in *M. robertsii*. Table S1: Primers of deletion, complementation and Construction of GFP fusion vector. Table S2: Gene expression analysis.
