# Supplementary figures and images for: Role of Two G-Protein α Subunits in Vegetative Growth, Cell Wall Integrity, and Virulence of the Entomopathogenic Fungus Metarhizium robertsii
Source: J Fungi (Basel). 2022 Jan 28;8(2):132. doi: 10.3390/jof8020132 (PMC8877820; doi:10.3390/jof8020132)

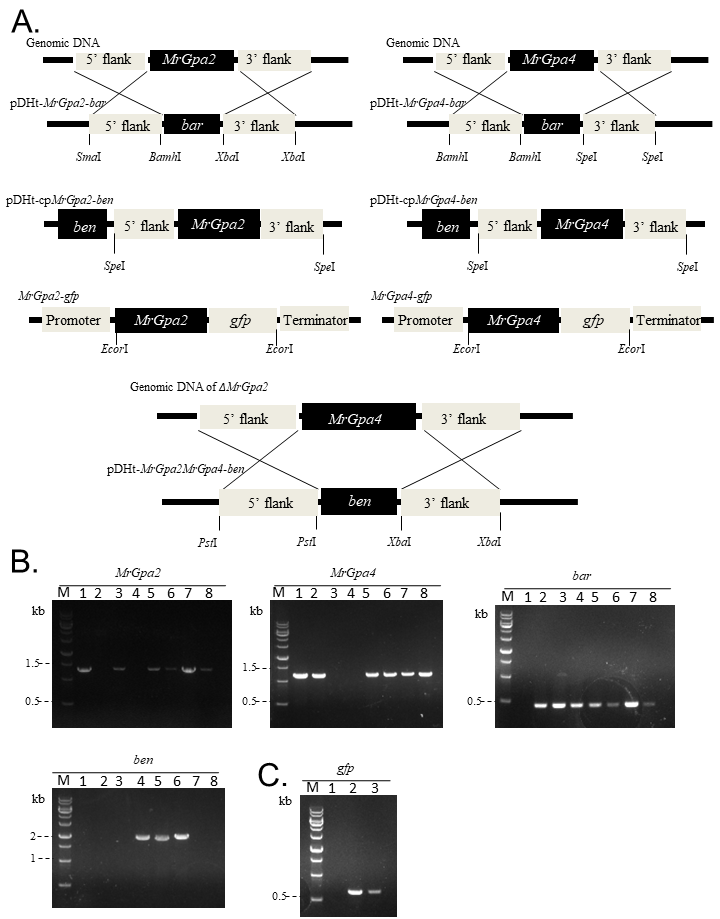

Supplement: Supplementary file 1 [file jof-08-00132-s001.zip › figure S1.tif]
